# Discovery of Pathologic GPCR Aggregation

**DOI:** 10.3389/fmed.2019.00009

**Published:** 2019-01-30

**Authors:** Ursula Quitterer, Said AbdAlla

**Affiliations:** ^1^Molecular Pharmacology, Department of Chemistry and Applied Biosciences, ETH Zurich, Zurich, Switzerland; ^2^Department of Medicine, Institute of Pharmacology and Toxicology, University of Zurich, Zurich, Switzerland

**Keywords:** G-protein-coupled receptor, oligomerization, preeclampsia, atherosclerosis, Alzheimer's disease, neurodegeneration, biased agonist, beta-arrestin

## Abstract

The family of G-protein-coupled receptors (GPCRs) is one of the most important drug targets. Mechanisms underlying GPCR activation and signaling are therefore of great pharmacologic interest. It was long thought that GPCRs exist and function as monomers. This feature was considered to distinguish GPCRs from other membrane receptors such as receptor tyrosine kinases or cytokine receptors, which signal from dimeric receptor complexes. But during the last two decades it was increasingly recognized that GPCRs can undergo aggregation to form dimers and higher order oligomers, resulting in homomeric and/or heteromeric protein complexes with different stoichiometries. Moreover, this protein complex formation could modify GPCR signaling and function. We contributed to this paradigm shift in GPCR pharmacology by the discovery of the first pathologic GPCR aggregation, which is the protein complex formation between the angiotensin II AT1 receptor and the bradykinin B2 receptor. Increased AT1-B2 heteromerization accounts for the angiotensin II hypersensitivity of pregnant women with preeclampsia hypertension. Since the discovery of AT1-B2, other pathologic GPCR aggregates were found, which contribute to atherosclerosis, neurodegeneration and Alzheimer's disease. As a result of our findings, pathologic GPCR aggregation appears as an independent and disease-specific process, which is increasingly considered as a novel target for pharmacologic intervention.

## Introduction

G-protein-coupled receptors (GPCRs) constitute one of the largest gene families in the human genome, and provide the target for about 20–30% of all drugs currently on the market. Since the discovery of rhodopsin and the beta-adrenergic receptor as first GPCRs (1, 2), currently more than 800 different genes are classified as members of the GPCR family. The pharmacologic and biologic importance of this huge receptor family led to enormous efforts world-wide to delineate activation and signaling mechanisms of GPCRs. A common theme of GPCRs is their seven membrane-spanning domain structure (3), which makes this type of protein a versatile platform for sensing of a panoply of different signals and stimuli including light, stress, hormones, peptides, proteins, ions, volatile odorants, tastants, and mechanical forces.

The seven membrane-spanning domain structure distinguishes GPCRs from other membrane receptors, which are, e.g., single-pass membrane proteins such as tyrosine kinase receptors or cytokine receptors (4). Consequently, major differences between GPCRs and these non-GPCR membrane receptors were found. The major distinguishing feature is that GPCRs signal through activation of heterotrimeric G-proteins, which gives the name to this class of receptors (5, 6).

Mechanistic studies of GPCR proteins focused on the identification of features, which account for GPCR-mediated G-protein activation. A major understanding came from experiments with receptor-derived peptides, which encompass segments of the second, third or fourth cytoplasmic domain of a GPCR and are capable to activate G-proteins independently of the presence of the entire transmembrane-spanning GPCR (7, 8). Other studies with purified receptor preparations found that interaction of a single GPCR with a single G-protein was a sufficient cause for G-protein activation (9, 10). Consequently, based on a 1:1 stoichiometry between a GPCR and a heterotrimeric G-protein, it was concluded that GPCRs function as monomers, and this feature distinguishes GPCRs from other membrane receptors such as tyrosine-kinase receptors or other monotopic transmembrane receptors, which require dimerization for receptor activation and signaling (4).

In contrast to these apparently conclusive data, many researchers, who worked with purified GPCRs, found protein aggregation of GPCRs *in vitro*, by gel filtration and on SDS-PAGE, and cooperative effects in various experimental settings. All these data could be interpreted as evidence for a dimeric/oligomeric assembly of GPCRs under certain experimental conditions. The first thematic study on GPCR dimerization was published in 1996 by the group of Michel Bouvier, who reported the functional characterization of GPCR dimers formed of beta-2-adrenergic receptors expressed in Sf9 cells (11). This was the beginning of a new era, and many other researchers started to report their “forbidden” findings of GPCR dimerization with transfected and endogenous receptors. But the question of relevance of GPCR dimerization still remained because most studies were performed with overexpressed receptors. There was the identification of the GABA(B) receptor (GABA-B; GABBR) heterodimer composed of GABBR1 and GABBR2, which as a class C GPCR relies on receptor dimerization for protein folding and function (12, 13). According to the A-F classification scheme of GPCRs, class C receptors contain a huge N-terminal clam-shaped domain with almost 600 amino acid residues (14, 15). This extracellular region of GABA-B receptors forms a dimer even in the absence of the transmembrane domain-containing protein core (15). Therefore, it was concluded that GABBR is a unique example of a GPCR that requires heterodimerization for functionality whereas for most other GPCRs, dimerization is a redundant process and has no obvious *in vivo* relevance.

**Figure 1:**
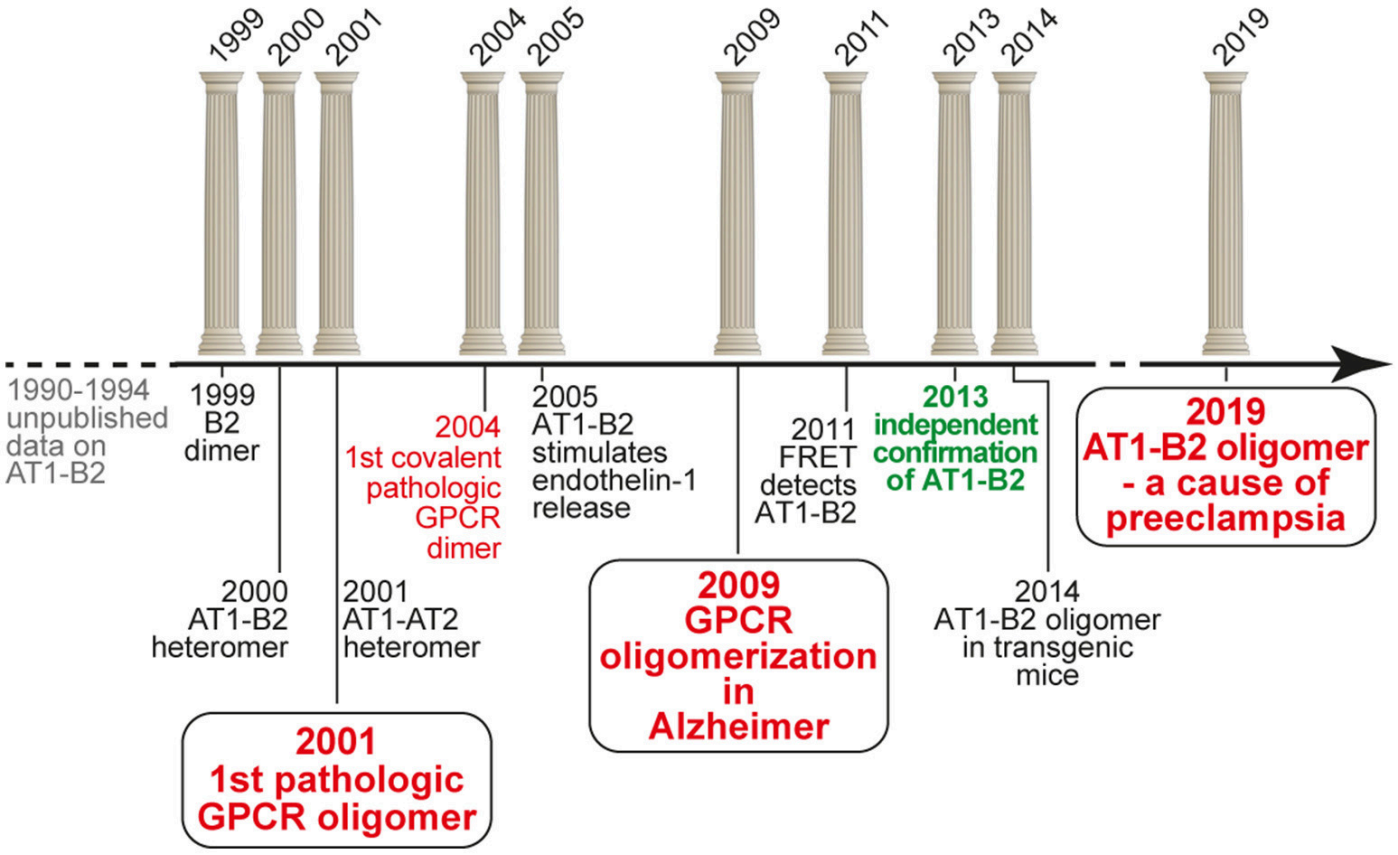
Timeline of the discovery of pathologic GPCR aggregation.

Beginning from 1999, we initiated an independent research project on GPCR homo- and heterodimerization, which was based on our previous unpublished data in the early nineties. And we discovered the first pathologically relevant GPCR hetero(di)mer, which is the protein complex formed between the vasopressor angiotensin II AT1 receptor and the vasodepressor bradykinin B2 receptor (16, 17). We found that increased protein complex formation between AT1 and B2 on circulating blood cells and vessels of patients with preeclampsia hypertension contributes to the angiotensin II hypersensitivity of this severe pregnancy-specific complication (17, 18). Our findings were intensely disputed (19), and final acceptance of our findings only came after more than a decade of scientific discussions (20). Meanwhile, our findings contributed to a paradigm shift in GPCR pharmacology, which is now on the verge to lead to new pharmacologic treatment approaches (21). The following article briefly overviews the timeline of the discovery of pathologic GPCR aggregation (Figure 1).

## First Evidence of an Interaction Between the Bradykinin B2 Receptor and the Angiotensin II AT1 Receptor in the Early 1990s (1990-1994)

Our discovery of a functional AT1-B2 receptor protein complex began in the early 1990s at the Institute of Pathobiochemistry of the University of Mainz (Germany), where our key findings were made during a project on the protein purification of the bradykinin B2 receptor from native human skin fibroblasts applying ligand and antibody affinity chromatography. This work was performed in frame of a common project between U. Quitterer and S. AbdAlla, who led the protein purification group. He found by amino-terminal protein sequencing that enrichment of the bradykinin B2 receptor protein, yielded co-enrichment of the angiotensin II AT1 receptor. At the same time, these data were complemented by U. Quitterer's functional studies, which showed that angiotensin II could induce a conformational change at the bradykinin B2 receptor with a shift of the B2 receptor from a high affinity to a low affinity binding state for the agonist bradykinin. This affinity shift could reflect a conformational change imposed by the activated AT1 receptor on the associated B2 receptor with subsequent G-protein activation and uncoupling of the B2 receptor, as a process which is known of other GPCRs to decrease agonist affinity (22, 23). “We discovered receptor heterodimers,” was the immediate interpretation of S. AbdAlla, who came from the Heinrich-Pette-Institute, Hamburg (Germany), where he had worked on dimerization of interleukin-2 and interleukin-2 receptors. But when we presented our data and the idea of AT1-B2 receptor dimerization to the Head of the Institute, he opposed this concept because in the GPCR field, protein dimerization does not exist. This was the end of our project on AT1-B2 receptor dimerization in Mainz (Figure 1).

## Discovery of AT1-B2 as the First Pathologic GPCR Heteromer (1999-2001)

After completion of the doctorate at the University of Mainz in 1994, and a postdoctorate at Roche Bioscience (Palo Alto, CA, USA) in 1995, U. Quitterer moved to the Institute of Pharmacology at the University of Wuerzburg (Germany) to continue research on GPCRs in 1996. Only 1 year later, in 1997, she re-established contacts with S. AbdAlla, who at that time became professor of biochemistry at the newly founded Genetic Engineering and Biotechnology Research Institute (GEBRI) in Alexandria, Egypt. We continued our work on AT1-B2 receptor heterodimerization, and during our studies, we discovered and characterized functional bradykinin B2 receptor homodimers in 1999 (24). The project on GPCR dimerization was conducted together with Dr. Heinz Lother, who invited S. AbdAlla as a visiting scientist into his labs at the Heinrich-Pette-Institute, Hamburg (Germany). After we had opened the path for the concept of receptor dimerization in the kinin field in 1999, the study on AT1-B2 receptor heteromerization was finished in a short time, and in the year 2000, we published the discovery of AT1-B2 receptor heteromerization in Nature [Figure 1; (16)].

**Figure 2:**
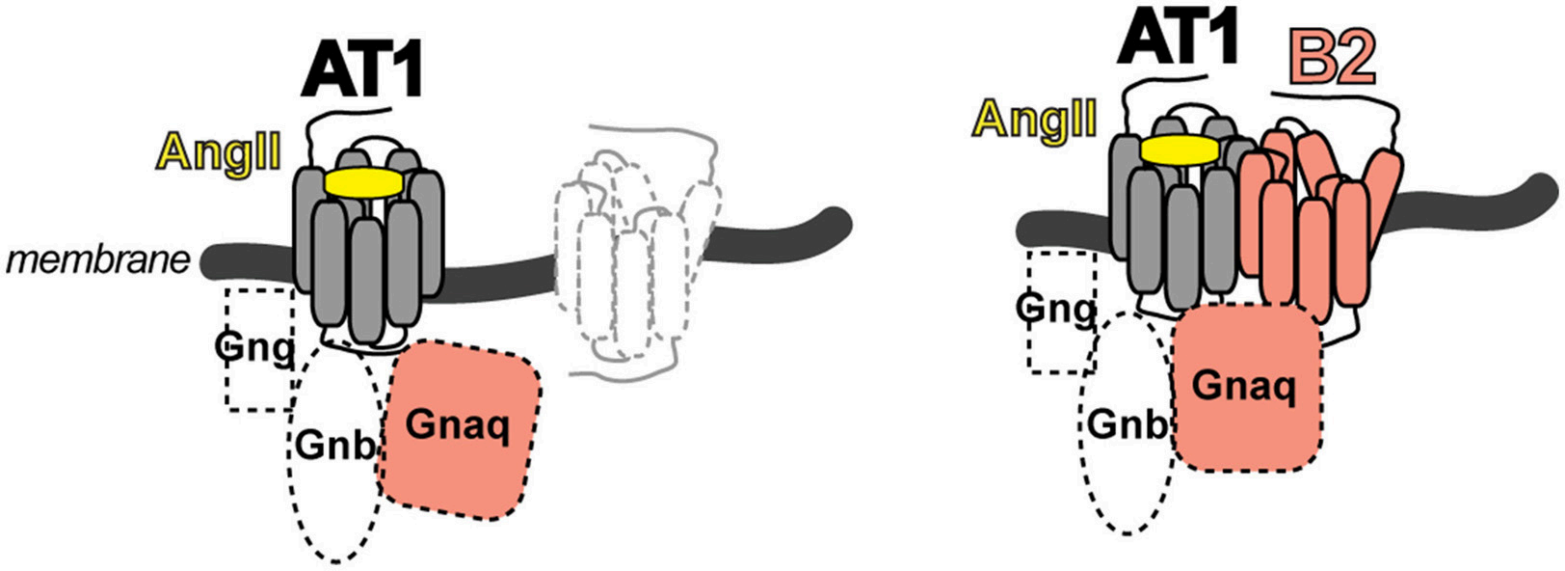
Scheme of the AT1-B2 heteromer, which forms a platform for enhanced G-protein activation **(right)** compared to a monomeric AT1 receptor **(left)**.

The identified heteromeric protein complex between AT1 and B2 receptors was functional, and led to enhanced angiotensin II AT1-mediated G-protein activation and signaling. The B2-mediated sensitization of the AT1 receptor-mediated response was independent of B2 receptor stimulation with the agonist bradykinin because a B2 receptor mutant with defective bradykinin binding still enhanced the AT1-stimulated G-protein activation and signaling. In contrast, a B2 receptor mutant with defective G-protein activation due to a mutation in the DRY motif in the 2nd intracellular loop of the B2 receptor was incapable to enhance AT1-stimulated signaling (16). These data are compatible with the notion that activation of the AT1 receptor by angiotensin II imposes a conformational change onto the associated unstimulated B2 receptor, which in turn adopts an active conformation capable to promote GDP-GTP exchange of the B2-coupled G-protein with subsequently enhanced signaling (Figure 2). The heteromeric AT1-B2 protein complex thus could constitute a platform, which enables enhanced G-protein activation (Figure 2).

The study of the AT1-B2 heteromer was complemented by our discovery of the AT1-AT2 receptor complex, which is a prototype of an inhibitory GPCR interaction [Figure 1; (25)]. In contrast to the B2 receptor, which enhances the angiotensin II AT1-stimulated G-protein activation (16, 17), the angiotensin II AT2 receptor inhibits the activation of the AT1 receptor by direct protein interaction (25). Thus, GPCR dimerization is capable to modulate the activation state of the dimerizing partner by direct interaction. As a consequence, dimerization could enhance or dampen the guanine nucleotide exchange factor-like function of a GPCR toward the heterotrimeric G-protein.

A major point in the field of GPCR dimerization and oligomerization always was the question about physiologic and pathophysiologic relevance. Most experiments on GPCR dimerization were made with transfected receptors and/or with cultured cells. Therefore, our focus immediately shifted to the question: Does AT1-B2 heteromerization occur *in vivo*? We knew that angiotensin II hypersensitivity was a major feature of preeclampsia hypertension (26, 27), which is the most frequent pregnancy-related complication with no cure. To study the role of AT1-B2 heteromerization in preeclampsia, S. AbdAlla initiated a collaboration with the Medical Research Center (MRC) at Ain Shams University, Cairo. The head of MRC, Prof. Adel el Missiery, strongly supported the project because preeclampsia has a high frequency among women in Egypt. Our study found that AT1-B2 contributes to the angiotensin II hypersensitivity of women with preeclampsia (17). This publication of “Receptor double-trouble in preeclampsia” (17, 18) marks the discovery of the first pathologic GPCR oligomer (Figure 1).

In this study, the AT1-B2 receptor heteromer was identified *in vivo*, after covalent stabilization with a cleavable cross-linker followed by affinity enrichment with AT1 receptor-specific antibodies and subsequent immunoblot detection of the co-enriched B2 receptor. This approach detected a significantly higher content of AT1-B2 heteromeric complexes on platelets and omental vessels from preeclampsia patients compared to biopsy specimens from uncomplicated pregnancies (17). Reciprocal experiments with enrichment of B2 and detection of co-enriched AT1 receptor gave similar results, and confirmed the disease-related AT1-B2 receptor aggregation in preeclampsia (17).

Our study also shows that augmented AT1-B2 receptor protein complex formation is disease-relevant and contributes to the well-established angiotensin II hypersensitivity in preeclampsia with enhanced angiotensin II-stimulated calcium signaling of platelets isolated from preeclamptic women (17, 26, 27). Sensitization of the angiotensin II-stimulated response is mediated by the AT1-B2 receptor heteromer not only in transfected cells but also in patients with preeclampsia (17). This conclusion was proved by domain-specific antibodies, which shielded the connecting loop between membrane domains III-IV of the bradykinin B2 receptor. With these antibodies, we found that the bradykinin B2 receptor protein is involved in angiotensin II-stimulated AT1-B2-mediated G-protein activation on omental vessels isolated from preeclamptic patients. Notably, the angiotensin II-stimulated response was largely blocked by these domain-specific antibodies to the connecting loop between membrane domains III-IV of the bradykinin B2 receptor whereas control antibodies had no effect (17). Together these findings complement our previous data on AT1-B2, which show that mutation of the DRY motif in the same connecting loop between membrane domains III-IV of the B2 receptor abolishes the B2-mediated sensitization of the angiotensin II-stimulated response triggered by the AT1-B2 heteromer (16).

In subsequent years, the pathophysiologic importance of aberrant AT1-B2 aggregation was supported by additional studies. Notably, AT1-B2 receptor heteromerization was found to enhance endothelin-1 release [Figure 1; (28)], which now is known as a major factor contributing to symptoms of preeclampsia (29).

## Discovery of Covalently Stabilized AT1 Receptor Dimers With Pathologic Relevance for Cardiovascular Disease and Atherosclerosis in 2004

After elucidation of the pathologic relevance of AT1-B2 receptor heteromerization, we continued our search for aberrant GPCR dimerization as contributor to pathomechanisms of disease. In view of our previous studies, we focused on disease states with angiotensin AT1 receptor hypersensitivity and found an increased content of covalently stabilized AT1 receptor dimers on monocytes isolated from the peripheral blood of patients with hypertension (30). This finding of stable AT1 dimerization on monocytes of hypertensive patients in the year 2004 marks the discovery of the first covalently stabilized GPCR dimer in human pathology [Figure 1; (30)]. The covalent stabilization of AT1 receptor dimers was unprecedented and mediated by an exaggerated intracellular transglutaminase factor XIIIA activity in patients with increased cardiovascular risk factors (30).

The crosslinking activity of the intracellular factor XIIIA does not rely on thrombin, which activates circulating factor XIIIA in plasma by proteolytic cleavage as part of the blood clotting cascade. Instead, the transglutaminase activity of intracellular factor XIIIA could be activated by an altered cellular ion homeostasis, increased cytosolic calcium and/or oxidative stress (30). All these causes of intracellular factor XIIIA transglutaminase activation are promoted by cardiovascular risk factors such as hypertension, hypercholesterolemia, and atherosclerosis. In concert with induction of factor XIIIA gene expression and protein in monocytes by cardiovascular risk factors (30), the consecutively exaggerated intracellular factor XIIIA activity accounts for covalent stabilization of AT1 receptor dimers, which are triggered by increased circulating angiotensin II levels in patients “at the onset of atherosclerosis” (30–32).

Our functional studies show that covalent stabilization of AT1 receptor dimers accounts for angiotensin II hypersensitivity *in vivo* and contributes to the atherogenic function of the AT1 receptor on monocytes of patients with cardiovascular disease and hypertension (30). Apart from disease relevance, our studies also confirm our previously established concept of modulation of G-protein activation by receptor dimerization: covalent stabilization of AT1 receptor homodimers creates a receptor platform, which enables efficient G-protein activation compared to AT1 receptor monomers and/or dissociable AT1 receptor complexes (30–32).

## Move to ETH Zurich and Discovery of Pathologic GPCR Aggregation in Alzheimer's Disease (2006–2009)

Our work on the discovery of pathologic GPCR aggregation was highly recognized by the international scientific community. The initial publication in Nature in the year 2000 (16) was ranked top 1% in the scientific field according to ISI web of science. Meanwhile this publication is cited more than 500 times. The discovery of AT1-B2 heteromerization as the first pathologic GPCR aggregation also became international textbook knowledge and was included in the “Blue Bible,” which is the nickname of the 12th edition of Goodman Gilman‘s The Pharmacological Basis of Therapeutics (33). Another highlight was the Wenner Grenn Symposium on GPCR dimerization in Stockholm in 2004. Kjell Fuxe from Karolinska Institute invited major players in the field of GPCR dimerization to Stockholm. During the meeting, U. Quitterer had the chance to dispute the existence and pathologic relevance of GPCR aggregation on stage with Robert Lefkowitz, who later received the Nobel Prize in Chemistry, in the year 2012, for studies on G-protein-coupled receptors. The discussion was moderated by Michel Bouvier, who supported U. Quitterer‘s statements and conclusions. With a high international reputation, the President of ETH Zurich, Prof. Olaf Kübler, offered U. Quitterer at the age of 39, the Chair of Molecular Pharmacology at ETH Zurich. She accepted the offer in summer 2005, and was nominated full professor and Chair of Molecular Pharmacology by the ETH Counsil at the end of 2005. We moved into newly refurbished ETH labs at the beginning of 2006.

At ETH Zurich, we continued our work on pathologic GPCR aggregation. Stimulated by ongoing discussions on existence and mechanisms of GPCR aggregation, our focus shifted to Alzheimer's disease (AD). We reasoned that GPCR aggregation should readily be detectable in a typical protein aggregation disease. We searched for covalently stabilized GPCR aggregates as a consequence or cause of Alzheimer senile plaques, because in a previous study, we found covalently stabilized GPCR aggregates formed of the angiotensin II AT1 receptor in atherosclerosis (30), which is also a “plaque-forming” disease. With this concept in mind, we discovered SDS-stable aggregates of the angiotensin II AT2 receptor as a characteristic feature of AD patient biopsy specimens isolated from prefrontal cortex (34, 35). Covalent aggregation of AT2 receptor oligomers is disease-specific for AD because related GPCRs such as the AT1 receptor are not aggregated in AD brains (34). The identification of covalently cross-linked AT2 receptor oligomers in brains of AD patients extends the pathologic relevance of aberrant GPCR aggregation to neurodegenerative diseases (Figure 1).

Why does the AT2 receptor form high molecular weight aggregates in brains of AD patients? We investigated the underlying mechanism and found that AT2 receptor aggregation in AD is a disease-specific process with two consecutive cross-linking steps, i.e., mediated by (i) reactive oxygen species (ROS), and (ii) transglutaminase. Initially, we aimed to deduce the AD-specific mechanism of AT2 aggregation in the transgenic Tg2576 AD model with neuron-specific expression of APP^Swe^, which is a mutant of the amyloid precursor protein (APP) isolated from a Swedish family with familial AD (36). Tg2576 AD mice reproduce major AD features such as symptoms of neuronal degeneration, cognitive impairment and beta-amyloid (Abeta) plaques in the hippocampus starting at an age of 12 months. However, Tg2576 mice did not develop pathologic AT2 oligomers but only showed the initial ROS-dependent AT2 crosslinking step, which leads to stable AT2 dimers (34). In search for the mechanism of pathologic AT2 aggregation in AD, we realized that aged Tg2576 AD mice are largely devoid of neuronal loss, which is a major characteristic of AD patients (34). To enhance the process of neurodegeneration, we subjected aged Tg2576 AD mice to chronic unpredictable mild stress (CUMS), which mimics psychosocial stress as an established risk factor for AD in patients (37). Chronic unpredictable mild stress augmented the hippocampal transglutaminase activity of Tg2576 mice, and triggered the final transglutaminase-mediated crosslinking of oxidized AT2 dimers to AD-related and pathologic AT2 oligomers. Concomitantly, chronic unpredictable mild stress increased hippocampal Abeta accumulation, promoted neurodegeneration-enhancing PHF tau phosphorylation and induced overt neuronal loss in the hippocampus of Tg2576 mice.

Cellular accumulation of covalently cross-linked AT2 receptor oligomers is pathologic and contributes to neurodegeneration and neuronal loss, which are hallmarks of AD brains. The pathologic AT2 receptor aggregates promote neuronal deterioration in AD by sequestration and inhibition of cognition-enhancing Gq/11-mediated-signaling stimulated, e.g., by the M1-cholinoceptor (34, 35). In agreement with this notion, inhibition of AT2 receptor oligomerization retarded symptoms of neurodegeneration in the experimental AD model (35). Our ongoing studies aim to translate these experimental findings into a therapeutic strategy to restore the function of the neuroprotective AT2 receptor and halt disease progression in AD models and patients (34, 35).

## Difficulties to Detect AT1-B2 Receptor Heteromerization

Immediately after the discovery of covalent AT2 receptor aggregation in AD (34, 35), the research on neurodegenerative GPCR aggregation in Alzheimer's disease was interrupted by a publication, which reported “lack of evidence for AT1R/B2R heterodimerization” (19). When we saw this and similar other publications (19, 38), we realized that we had to redirect our research toward the AT1-B2 heteromer.

In search for causes, which could account for the failure to detect the AT1-B2 receptor heteromer in transfected cells, we focused on the role of chaperones because chaperones are critically influenced by variable cell culture conditions (39). Whole genome microarray gene expression profiling identified the chaperone, calreticulin, as an important factor required for efficient B2 receptor maturation and AT1-B2 heteromerization (39). Loss of essential chaperones during *in vitro* cell culture could be a cause for failure to detect AT1-B2 (39). To overcome limitations of *in vitro* cell culture, we established cell expansion conditions, which mimic the *in vivo* environment because our studies mainly focus on GPCR aggregation under pathologic conditions *in vivo*. The *in vivo* cell expansion model applies NOD.Scid mice for expansion of cultured cells. Microarray gene expression analysis documented that the expression of essential chaperones is restored upon *in vivo* expansion of cultured cells in NOD.Scid mice (40).

Another critical factor is the intrinsic pathologic nature of the AT1-B2 heteromer, which could contribute to further difficulties to detect AT1-B2 in transfected cells. Expression of functional AT1-B2 heteromers leads to exaggerated calcium signaling with consecutively reduced cellular growth rates, premature senescence, and ultimately calcium-induced cell death (41, 42). Consequently, only slow-growing cells with low AT1-B2 expression level will survive. Under these conditions, there is a high risk that the cell culture dish is rapidly overgrown by fast dividing, non-transfected cells and/or cells expressing either AT1 or B2. This scenario is particularly relevant for *in vitro* cultured cells due to the loss of essential pro-survival factors, e.g., components of the pro-survival ERK pathway (43). Control experiments, which control for co-expression of AT1-B2 at the single cell level are necessary to identify and overcome such problems.

At present, it is not clear, why several groups failed to detect AT1-B2 receptor heteromerization in transfected cells. While we addressed some possible issues, a definite answer is not possible because experiments in these publications were performed with batches of cells, and precise biochemical and/or histological control experiments are lacking, e.g., the control for co-expression of AT1 and B2 at the single cell level, co-immunoenrichment studies and immune-fluorescent microscopy of receptor proteins (19, 38).

Apart from the above-mentioned causes, a high cellular expression level of the transfected GPCRs, notably the AT1 receptor, could become another factor of failure to detect AT1-B2 heteromerization. The documented reporting of constitutive AT1 receptor homomerization could be indicative of high AT1 receptor expression at the single cell level (19). High AT1 receptor expression at the single cell level could be attributed, e.g., to the SV40 large T-antigen-driven DNA replication at high copy number in COS cells (19). A high cellular GPCR expression level could be a critical factor because it could favor the formation of receptor homomers over receptor heteromers (44), i.e., the homomeric interaction between AT1-AT1 could become dominant and prevent the heteromeric interaction of AT1 with B2. As mentioned above, essential control experiments were not performed, e.g., the visualization of AT1 and B2 receptor proteins by immunoblot to detect protein aggregation and/or immaturely folded proteins (19, 38).

Concomitantly with the identification of critical parameters required for AT1-B2 heteromer formation in cells, we established a versatile method for detection of the AT1-B2 receptor heteromer by confocal FRET imaging, which is capable to detect protein-protein interactions in close proximity at a distance of < 10 nm (45). We added a signal peptide to the AT1 receptor-cerulean fusion protein, which otherwise barely reached the cell surface under standard cell culture conditions. With this approach, we demonstrated a high FRET efficiency of 24.7% for the interaction between AT1-Cerulean and B2-eYFP (45). Thus, we had proven that the AT1-B2 receptor heteromer exists in cultured cells, and can readily be detected by a standard FRET-based method, which is usually applied for detection of GPCR dimerization in cells (Figure 1). In addition, we documented functionality of AT1-B2 receptor heteromerization, which accounts for receptor co-internalization upon stimulation with angiotensin II (45).

Taken together, the question, why several groups were unable to detect AT1-B2 receptor heteromerization in cells, cannot be answered because specific control experiments were not performed (19, 38). In contrast, different groups in different laboratories worldwide demonstrated that AT1-B2 receptor heteromerization can be detected by different biochemical and biophysical methods (16, 17, 20, 45).

## Independent Confirmation of AT1-B2 Heteromerization in the Year 2013, after more than a Decade of Scientific Discussions

While our own work established the methodology for detection of functional AT1-B2 heteromerization, final acceptance of AT1-B2 by the scientific community occurred in 2013, after more than a decade of scientific discussions (Figure 1). In 2013, the group of Louis Luttrell, a former member of the Nobel Prize-winning Lefkowitz lab, reproduced our findings of AT1-B2 receptor heteromerization (20). With this publication, years of ongoing scientific discussions were finally resolved. The publication not only confirmed our initial findings but also opened the path to a new pharmacologic targeting approach of the AT1-B2 heteromer by the beta-arrestin-biased AT1 agonist, SII (Sar1,Ile4,Ile8-AngII) (20).

**Figure 3:**
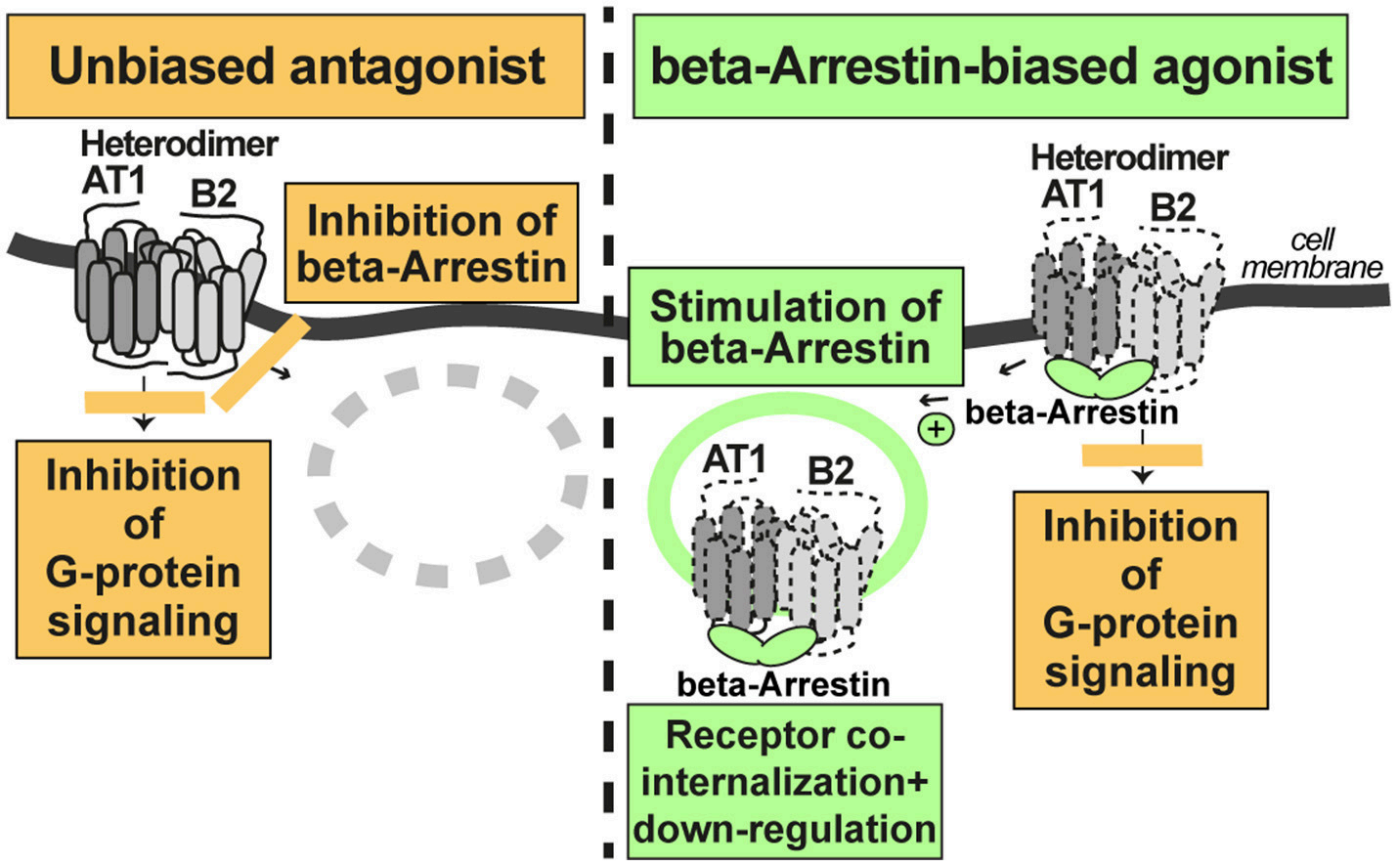
A beta-arretin-biased agonist targets the AT1-B2 heteromer and promotes AT1-B2 down-regulation **(right)**. The unbiased AT1 antagonist does not promote AT1-B2 down-regulation **(left)**.

According to the concept of beta-arrestin-biased agonism, stimulation of the AT1 receptor by a beta-arrestin-biased agonist inhibits G-protein-stimulated signaling but promotes the recruitment of beta-arrestin to the activated AT1-B2 receptor complex. As a consequence of beta-arrestin recruitment, the AT1-B2 receptor complex undergoes co-internalization and subsequent down-regulation (Figure 3).

In contrast, a classic, unbiased AT1 receptor antagonist such as losartan, blocks both, AT1-stimulated signaling, and beta-arrestin recruitment to the receptor. Consequently, the AT1-B2 receptor complex is not down-regulated (Figure 3). All currently approved AT1 antagonists are unbiased and contraindicated in preeclampsia because they cross the placental barrier and promote fetal malformations in pregnancy (46). Experimental beta-arrestin-biased agonists such as SII or TRV027 are peptides (47, 48) and not expected to cross the placental barrier. Therefore, such beta-arrestin-biased AT1 agonists could be envisaged as a future therapy for preeclampsia. Notably, beta-arrestin-biased AT1 agonists target the AT1-B2 receptor heteromer and promote AT1-B2 heteromer co-internalization and subsequent down-regulation (20, 49, 50).

## Outlook

Our ongoing studies at ETH Zurich currently address the feasibility to target the AT1-B2 heteromer and other pathologic GPCR aggregates by beta-arrestin-mediated down-regulation. To study such an approach, we generated transgenic models with increased AT1-B2 heteromerization. With these transgenic models, the *in vivo* relevance of AT1-B2 receptor heteromerization was demonstrated (50). Our data show that increased vascular AT1-B2 levels are a sufficient cause for preeclampsia symptoms in pregnant mice [Figure 1; (50)]. Moreover, symptoms of preeclampsia in AT1-B2-transgenic mice can be prevented by beta-arrestin-mediated down-regulation of AT1-B2 (50). Thus, targeting of the AT1-B2 heteromer by beta-arrestin-mediated down-regulation is feasible (50). And the initial identification of pathologic GPCR aggregation more than a decade ago is currently being translated into a new pharmacologic treatment approach.

## Author Contributions

UQ and SA wrote, read, and approved the final version of the manuscript.

### Conflict of Interest Statement

The authors declare that the research was conducted in the absence of any commercial or financial relationships that could be construed as a potential conflict of interest.
